# Influence of α-Calcium Sulfate Hemihydrate on Setting, Compressive Strength, and Shrinkage Strain of Cement Mortar

**DOI:** 10.3390/ma12010163

**Published:** 2019-01-07

**Authors:** Bokyeong Lee, Gyuyong Kim, Jeongsoo Nam, Kyehyouk Lee, Gyeongtae Kim, Sangkyu Lee, Kyoungsu Shin, Tomoyuki Koyama

**Affiliations:** 1 Mineral Resources Research Division, Korea Institute of Geoscience and Mineral Resources, Daejeon 34132, Korea; leebk@kigam.re.kr; 2 Department of Architectural Engineering, Chungnam National University, Daejeon 34134, Korea; j.nam@cnu.ac.kr (J.N.); kahn119@naver.com (G.K.); lsg2357@naver.com (S.L.); 3 RDM Industrial Development Co., Ltd., Daejeon 34831, Korea; ya-nalang@hanmail.net; 4 Corporate Research Institute, Yusung-Tech Co., Ltd., Okcheon 29057, Korea; shinks82@naver.com; 5 Department of Architecture and Urban Design, Kyushu University, Fukuoka 812-0053, Japan; koyama@arch.kyushu-u.ac.jp

**Keywords:** α-calcium sulfate hemihydrate, cement mortar, ettringite, initial setting time, compressive strength, shrinkage strain

## Abstract

This study focused on the quick initial setting time and the expansion strain that occurs during the early aging of α-calcium sulfate hemihydrate (αHH) and examined the setting, compressive strength, and shrinkage strain of αHH-replaced cement mortar. The results show that the initial setting time significantly decreased with an increase in the αHH replacement ratio. Drastic occurrence of ettringite was observed early in the aging of cement mortar when αHH was substituted into the cement; however, the ettringite was not converted to monosulfate with increasing age and thus was not favorable for the development of the compressive strength. When αHH was substituted into cement, using Portland blast-furnace slag cement (PSC) was more advantageous than using ordinary Portland cement (OPC) for the development of the compressive strength. Meanwhile, the expansion of early age αHH can decrease the shrinkage strain of cement mortar. The generation of ettringite is more effective when αHH is substituted into PSC than into OPC and is thus more effective in suppressing the shrinkage strain.

## 1. Introduction

Gypsum is a material in which the amount of water of crystallization varies with its surrounding conditions, such as temperature or humidity, and can be classified into three groups, namely, calcium sulfate anhydrite (CaSO_4_), calcium sulfate hemihydrate (CaSO_4_∙1/2H_2_O), and calcium sulfate dihydrate (CaSO_4_∙2H_2_O) [1]. Calcium sulfate hemihydrate can be further categorized into α and β forms, while calcium sulfate anhydrite can be further categorized into I, II, and III forms.

Previous studies have focused on the production of calcium sulfate hemihydrate from calcium sulfate anhydrite and calcium sulfate dihydrate [2,3,4,5,6]. Both α-calcium sulfate hemihydrate (αHH) and β-calcium sulfate hemihydrate are soluble gypsums that are easily hydrated via the surrounding moisture, and they characteristically hydrate and set into calcium sulfate dihydrate in the presence of water. The solubility of calcium sulfate hemihydrate is higher than that of calcium sulfate anhydrite or calcium sulfate dihydrate; hence, excessive additions result in supersaturated solutions, which lead to the precipitation and setting of calcium sulfate dihydrate. As previously mentioned, soluble gypsum calcium sulfate hemihydrate can easily absorb moisture and thus accelerate the rate of expansion and hardening.

**Figure 1:**
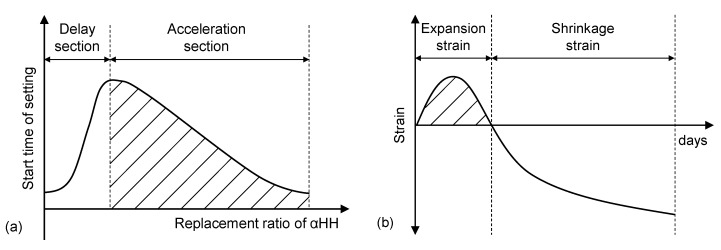
Conceptual diagram of (**a**) the start time of setting and (**b**) the strain properties of α-calcium sulfate hemihydrate (αHH).

Figure 1 shows a conceptual diagram of the initial setting time and strain properties of αHH. In general, the setting time of calcium sulfate dihydrate becomes almost constant above a certain rate of addition. The setting of αHH is delayed by additions of up to approximately 2.0%, but further additions result in the immediate precipitation of calcium sulfate dihydrate from the supersaturated solution and, subsequently, a shorter setting time [7]. In addition, the growth pressure of the acicular ettringite crystals formed from the reactions between C_3_A and αHH increases the distance between the cement particles or hydrates, resulting in expansion strains early in the aging process.

αHH is presently made from calcium sulfate dihydrate using an autoclave via pressurized steam or pressurized solution, or without an autoclave via atmospheric steam or atmospheric solution. However, due to the complex manufacturing steps, difficulties in mass production, and subsequent increase in material costs, these methods have not been very useful. On the other hand, the recent commercialization of a cost-effective αHH production technology that utilizes flue gas desulfurization gypsum has led to increased usability of αHH as a construction material [8,9,10,11].

αHH sets and hardens like cement when mixed with the appropriate amount of water. It has been reported that the strength of αHH is affected by its pore structure, the size and shape of its crystals, and the interactions among its microstructures [6]. Tang et al. [12] investigated the effects of different crystal sizes and shapes of αHH on its strength and microstructure. Guan et al. [13] and Ye et al. [14] evaluated the effects of particle size on the compressive strength of αHH. αHH significantly hardens at an early age, and after reaching maximum strength, the strength shows minimal change or a decreasing trend; this has been verified in previous studies [15,16]. Lewry and Williamson [17] claimed that the strength development of calcium sulfate hemihydrate occurs as a three-step process, and Guan et al. [15] used this trend to propose a five-step hydration process of αHH, namely, the bonding of particles in paste, formation of a crystal matrix, release of internal stress, evaporation of free water, and joining of the instability matrix [17,18,19]. In other words, it can be inferred that αHH is converted to calcium sulfate dihydrate via hydration, and the structure is loosened and the strength decreases with age due to recrystallization of the precipitated calcium sulfate dihydrate crystals.

Therefore, the objective of this study is to determine fundamental data for the application of αHH as a construction material. The study focused on the quick initial setting time and the expansion strain that occurs during the early aging of αHH and examined the setting, compressive strength, and shrinkage strain of αHH-replaced cement mortar. In addition, the crystalline structure and microstructure of αHH-replaced cement mortar were analyzed using X-ray diffraction (XRD), quantitative X-ray diffraction (QXRD), and scanning electron microscope (SEM) observations.

## 2. Experimental Procedures

### 2.1. Materials

Table 1 lists the physical properties, while Table 2 presents the chemical compositions of the materials used in the experiment. To produce αHH-replaced cement mortar, this study used ordinary Portland cement (OPC) and Portland blast-furnace slag cement (PSC). Type I OPC was used according to the Standard Specification for Portland Cement (ASTM C 150). The PSC consisted of 62% OPC and 38% ground granulated blast-furnace slag (GGBS). The αHH used in the experiment was derived from flue gas desulfurization gypsum using pressurized solution. For the fine aggregate, ISO standard sand was used.

**Figure 2:**
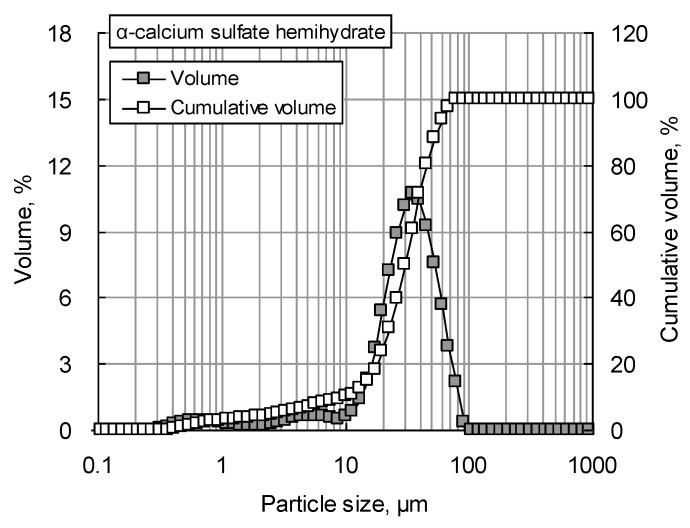
Particle size distribution of αHH.

**Figure 3:**
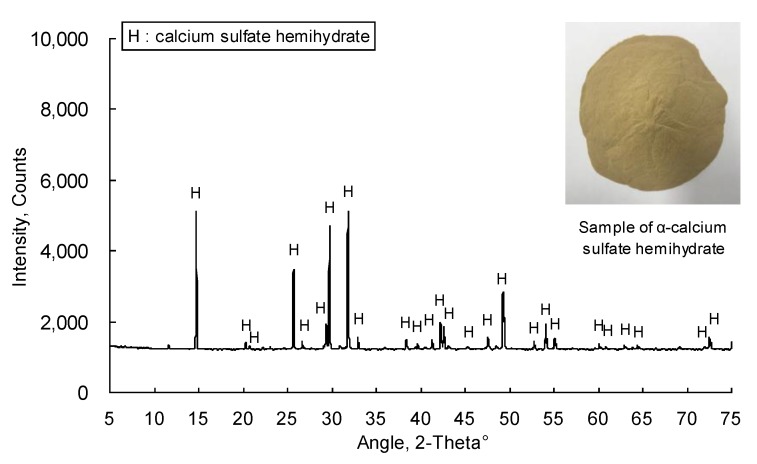
X-ray diffraction patterns of αHH.

**Figure 4:**
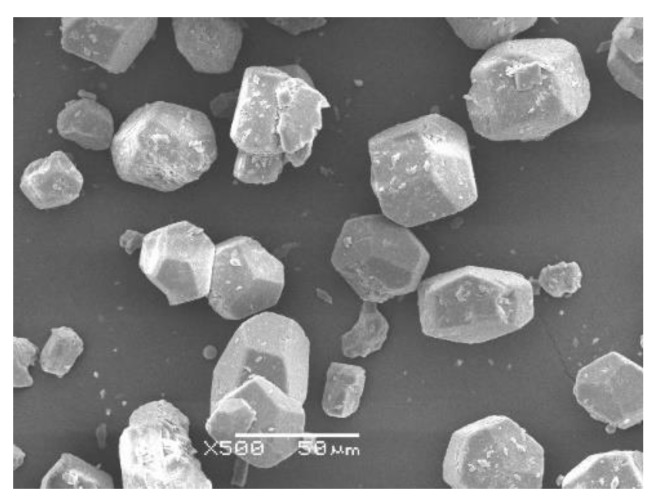
Scanning electron microscope micrograph of αHH.

Figure 2 shows the particle size distribution of αHH measured using a Mastersizer 2000 particle size analyzer (Malvern Instruments Ltd., Malvern, UK). The D_50_ of the αHH used in the experiment was 34.6 µm, while the D_90_ was 62.8 µm. The XRD patterns of the αHH are displayed in Figure 3. It was observed that the diffraction pattern of αHH includes most of the calcium sulfate hemihydrate peaks. Figure 4 shows a SEM micrograph of αHH.

### 2.2. Experimental Plan

The experimental plan of this study is summarized in Table 3. To evaluate the setting, compressive strength, and shrinkage strain of αHH-replaced cement mortar, the type of cement and the αHH replacement ratio were selected as the experimental variables. The experiment was conducted using αHH replacement ratios of 0, 10, 20, and 30 wt % in OPC, as well as 0 and 10 wt % in PSC. The setting time and compressive strength were measured along with the drying shrinkage to assess the shrinkage strain. In addition, XRD and QXRD were used to analyze the crystalline structure, while SEM micrographs were used to analyze the microstructure. The mortar was mixed according to the Test Methods for the Determination of Strength in Cement (ISO 679) with a water-to-binder weight ratio (W/B) of 0.5 and a binder-to-sand weight ratio (B:S) of 1:3.

### 2.3. Methods

The setting time was measured according to the Standard Test Method for Time of Setting of Concrete Mixtures by Penetration Resistance (ASTM C 403/C 403M), and regression analysis was used to determine the initial setting time and final setting time as the times when the penetration resistances were 3.5 MPa and 27.6 MPa, respectively.

The Standard Test Method for Compressive Strength of Hydraulic-Cement Mortars (ASTM C 349) was used to measure the compressive strength. Three specimens were fabricated for each age, each with dimensions of 40 × 40 × 160 mm^3^. The specimens were water cured at 20 ± 3 °C. To measure the compressive strength of each specimen, a dry surface was created by wiping away moisture and removing debris, and a mortar jig was used at ages of 1, 3, 7, and 28 days. A universal testing machine with a capacity of 2000 kN was used.

Each specimen used for the measurement of the drying shrinkage was fabricated with dimensions of 100 × 100 × 400 mm^3^ and an embedded dual flange strain gauge. After demolding, the strains were measured using a data logger in a room with constant temperature and humidity of 20 ± 3 °C and 60 ± 3%, respectively.

To analyze the crystalline structure of each specimen, XRD analysis (D/MAX-2200 Ultima/PC, Rigaku International Corporation, Tokyo, Japan) and QXRD were carried out at ages of 3 and 28 days by collecting each sample, processing it into powder, carrying out pretreatment, and subsequently taking measurements. XRD analysis was carried out using a Cu target and a D8 Advance diffractometer (Bruker-AXS, Karlsruhe, Germany) equipped with a LynxEye position-sensitive detector. The diffraction patterns were obtained at 2θ from 5° to 65° in steps of 0.01°, with each step lasting 1 s. The divergence slit was 0.3°, while the soller slit was 2.5°.

The microstructure of each specimen was observed using SEM (JSM-6380, Jeol Ltd., Tokyo, Japan) at ages of 3 and 28 days by coating each specimen with platinum and applying an accelerating voltage of 15 kV. The crystalline structure and microstructure analyses were carried out on the OPC specimen, as well as the OPC-αHH20 and PSC-αHH10 specimens, which had the least strains due to drying shrinkage.

## 3. Experimental Results and Discussion

### 3.1. Setting Time

**Figure 5:**
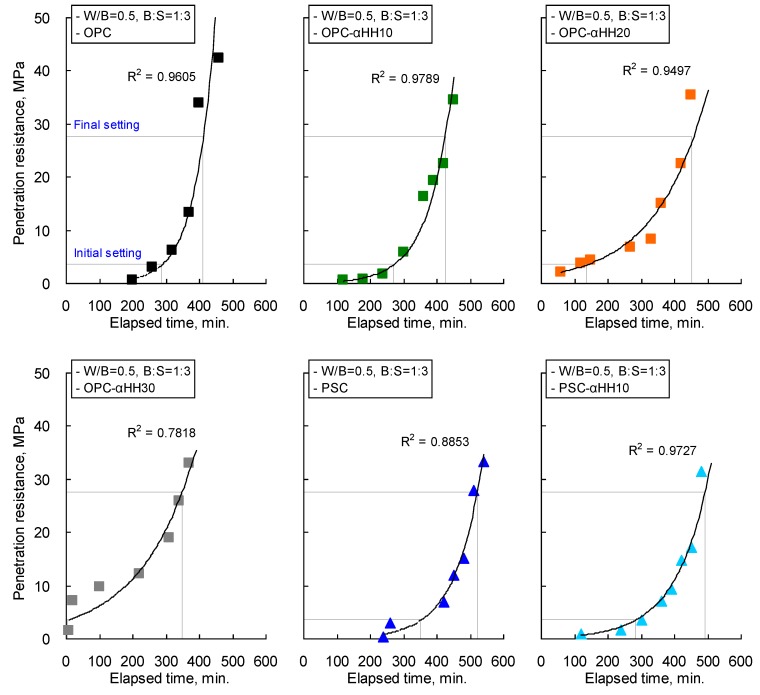
Penetration resistance measurement results of αHH-replaced cement mortar.

The penetration resistance measurement results of cement mortar with αHH are shown in Figure 5, and the setting times obtained from regression analysis are presented in Table 4. When OPC was used, the setting time decreased with increasing replacement ratios of αHH. The lowest final setting time occurred in the OPC-αHH30 specimen, and unlike the trends observed in the initial setting time, the final setting times of the OPC-αHH10 and OPC-αHH20 specimens appeared to be longer than that of the OPC specimen. The initial setting time of the OPC-αHH30 specimen, which had an αHH replacement of 30 wt %, was 6 min, which is significantly lower than that of the OPC specimen without αHH replacement, while the final setting time was 350 min, thus confirming the trend of increasing time between the initial setting and the final setting.

The coefficient of determination of the OPC-αHH30 specimen was calculated to be 0.7818, and the goodness of fit derived from regression analysis was relatively lower than those of other specimens. This trend is believed to be due to quick setting owing to excessive replacement of αHH.

In the case of the PSC specimen, the initial setting time and the final setting time were longer than those of the OPC specimen due to the latent hydraulic property of the GGBS. The PSC-αHH10 specimen, which had an αHH replacement of 10 wt %, had a significantly lower initial setting time and final setting time compared to those of the PSC specimen. However, the PSC-αHH10 specimen had an initial setting time similar to that of the OPC specimen, while its final setting time was found to be longer.

Meanwhile, the coefficient of determination was 0.8853 due to delayed setting of the PSC specimen because of the latent hydraulic property of the GGBS, while the goodness of fit was relatively lower than that of the OPC specimen. However, the coefficient of determination of the PSC-αHH10 specimen was 0.9727, indicating that the reduced setting time achieved by mixing αHH resulted in a goodness of fit that was greater than that of the PSC specimen.

At an αHH replacement ratio of 10 wt % in cement mortar, setting occurs faster in OPC than in PSC. However, the rate of reduction of the setting time was higher when αHH was substituted into PSC. These results indicate that αHH affected the activation of the hydration reaction more in PSC than in OPC.

### 3.2. Compressive Strength

**Figure 6:**
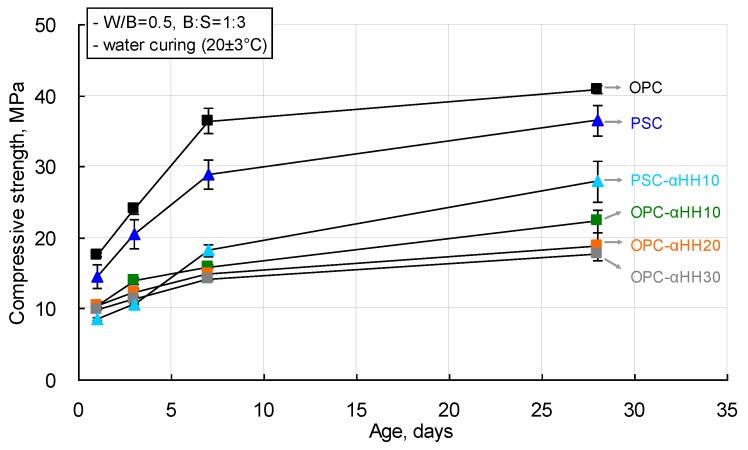
Compressive strength measurement results of αHH-replaced cement mortar.

The compressive strength measurement results of cement mortar with αHH are shown in Figure 6. The compressive strength of the cement mortar decreased with increasing replacement ratios of αHH. When OPC was used, the compressive strengths of the OPC-αHH10, OPC-αHH20, and OPC-αHH30 specimens at the age of 1 day were 10.43 MPa, 10.39 MPa, and 9.96 MPa, respectively, which are approximately 57–59% that of the OPC specimen (17.62 MPa). At 28 days, the compressive strengths of the OPC-αHH10, OPC-αHH20, and OPC-αHH30 specimens were 22.31 MPa, 18.79 MPa, and 17.81 MPa, respectively, which are approximately 44–55% that of the OPC specimen (40.88 MPa).

When PSC was used, the compressive strength of the PSC specimen was 14.54 MPa at the age of 1 day and 36.51 MPa at the age of 28 days. The compressive strength of the PSC-αHH10 specimen was measured to be 8.56 MPa—the lowest observed compressive strength at an early age in this study. These results indicate that αHH did not have a significant effect as a stimulant for the hydration of PSC at an early age. However, the compressive strength of the PSC-αHH10 specimen at the age of 28 days was 27.90 MPa, which is approximately 68% that of the OPC specimen. Therefore, among the cement mortars with αHH replacements, the PSC-αHH10 specimen, which used PSC, was confirmed to be most favorable for the development of the compressive strength.

Overall, replacement with αHH resulted in a decrease in the compressive strength in cement mortar, which was also reported in a previous study [16]. Meanwhile, a decrease in compressive strength owing to the use of only αHH was not observed within the αHH replacement ratio range used in the current experiment.

### 3.3. Drying Shrinkage

**Figure 7:**
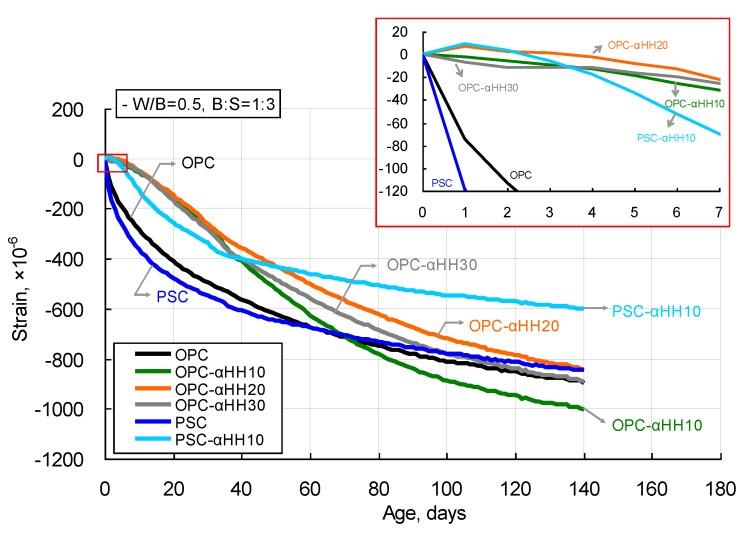
Drying shrinkage measurement results of αHH-replaced cement mortar.

Figure 7 shows the drying shrinkage measurement results of cement mortar with αHH. The OPC and PSC specimens that did not have αHH replacements showed drastic increases in shrinkage strains at their early ages, with the slope of the curve decreasing with time. However, when αHH was substituted in, the drastic increase in the shrinkage strain observed in the OPC and PSC specimens did not occur. This can be explained by the growth pressure of the acicular ettringite crystals formed from mixing with αHH, which increases the distance between the cement particles or hydrates, resulting in expansion strains in the early ages.

When OPC was used with αHH replacement, the strain curve appeared to be linear up to the age of approximately 40 days, with the slope gradually decreasing afterward. In particular, the OPC-αHH20 specimen displayed expansion behavior at an early age. The shrinkage strain at the early age of the αHH-replaced cement mortar was relatively less than that of cement mortar but increased with progressing age, while some specimens even displayed shrinkage strains that were greater than that of cement mortar.

The PSC-αHH10 specimen, which had αHH replacement in PSC, showed expansion behavior at its early age, as can be observed in the OPC-αHH20 specimen. The PSC-αHH10 specimen showed a more drastic increase in shrinkage strain than did the OPC-αHH10, OPC-αHH20, and OPC-αHH30 specimens; however, the slope of the strain curve decreased with age, resulting in the least shrinkage strain in the current experiment. Therefore, it was determined that replacing αHH in PSC was more effective for suppressing the shrinkage strain than replacing αHH in OPC.

### 3.4. X-ray Diffraction and Quantitative X-ray Diffraction Analysis

**Figure 8:**
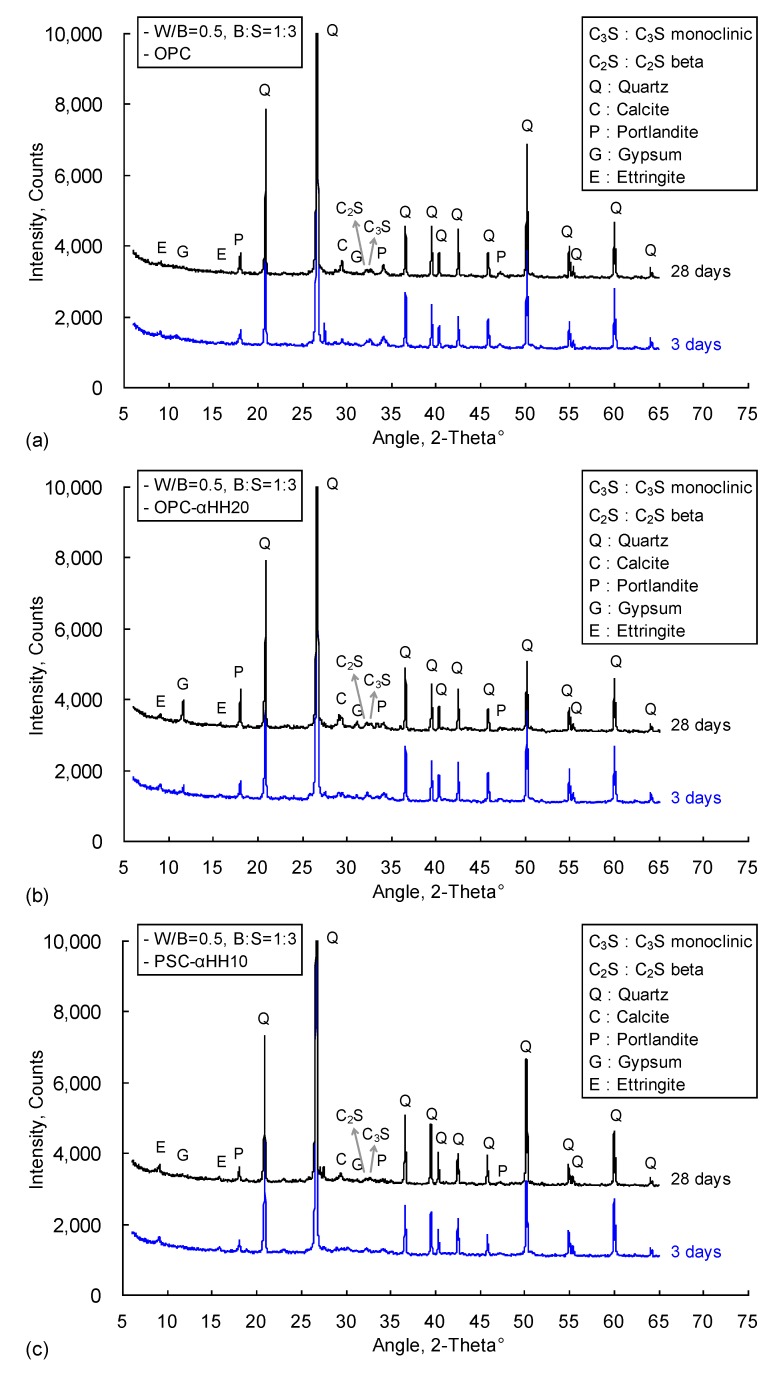
X-ray diffraction analysis of (**a**) OPC, (**b**) OPC-αHH20, and (**c**) PSC-αHH10 specimens.

Figure 8 shows the XRD analysis results of the OPC, OPC-αHH20, and PSC-αHH10 specimens at the ages of 3 and 28 days. Quartz peaks with high intensities were observed due to the mixing of fine aggregate, and similar diffraction patterns were observed in the specimens, including the occurrence of portlandite and ettringite. The intensity of the gypsum peak in the αHH-replaced OPC-αHH20 specimen was higher than those of the other specimens, and it increased with progressing age.

Table 5 presents the QXRD results of the OPC, OPC-αHH20, and PSC-αHH10 specimens at the ages of 3 and 28 days. As mentioned above, the OPC, OPC-αHH20, and PSC-αHH10 specimens all contained 83–86% quartz at the age of 3 days owing to the mixing of fine aggregate, and the quartz contents gradually decreased with progressing age. On the other hand, the αHH-replaced OPC-αHH20 and PSC-αHH10 specimens showed increasing ettringite contents compared to the OPC specimen. In particular, it was observed that the PSC-αHH10 had the highest ettringite content. Overall, the ettringite contents decreased with time, being less at the age of 28 days than at the age of 3 days. It is clear that replacement with αHH and the subsequent drastic production of ettringite at an early age accelerated the hydration reaction and decreased the setting time; however, the residual ettringite that was not converted to monosulfate, even with progressing time, appeared to have negatively impacted the development of the compressive strength.

As mentioned in the introduction, the growth pressure of the acicular ettringite crystals formed from the reactions between C_3_A and αHH increases the distance between the cement particles or hydrates, resulting in expansion strains early in the aging process. In other words, replacement with αHH can suppress the shrinkage strain in cement mortar, and this effect is believed to be at a maximum at the early age of the material. However, as confirmed from the QXRD results, the ettringite content decreases with age, which diminishes the effects of suppressing the shrinkage strain. Therefore, as shown in Figure 7, even with replacement with αHH, the effect of suppressing the shrinkage strain was not very significant with age progression. Consequently, when αHH was substituted into PSC, the production of ettringite was higher than in OPC; thus, the suppression of the shrinkage strain was more effective.

### 3.5. Scanning Electron Microscope Micrographs

**Figure 9:**
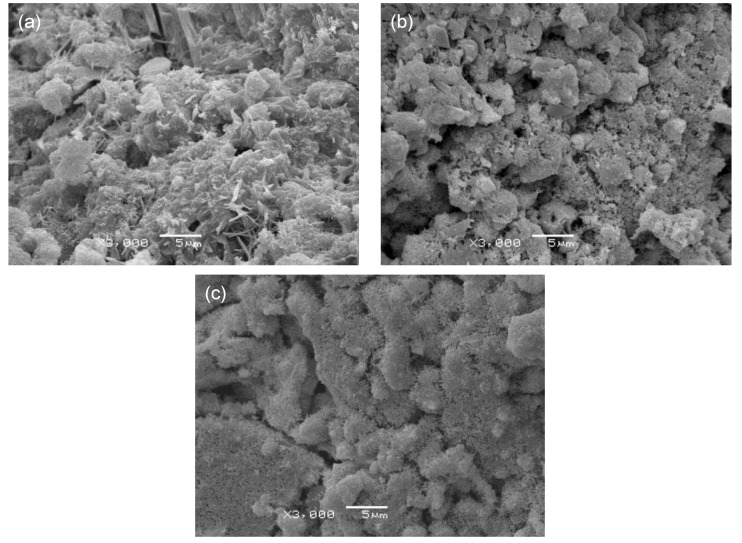
Scanning electron microscope micrographs of (**a**) OPC, (**b**) OPC-αHH20, and (**c**) PSC-αHH10 specimens at age of 3 days.

Figure 9 shows SEM micrographs of the OPC, OPC-αHH20, and PSC-αHH10 specimens at the age of 3 days. Compared to the OPC specimen, the αHH-replaced OPC-αHH20 and PSC-αHH10 specimens had wider distributions of acicular ettringite crystals. In general, ettringite causes the internal structure to become dilatated when widely distributed within the matrix and creates a negative effect of drastically decreasing the compressive strength when αHH is substituted into the cement. Moreover, as previously mentioned, the growth pressure of the ettringite acicular crystals increases the distance between the cement particles or hydrates; thus, replacement with αHH is effective for suppressing the shrinkage strain early in the aging process.

**Figure 10:**
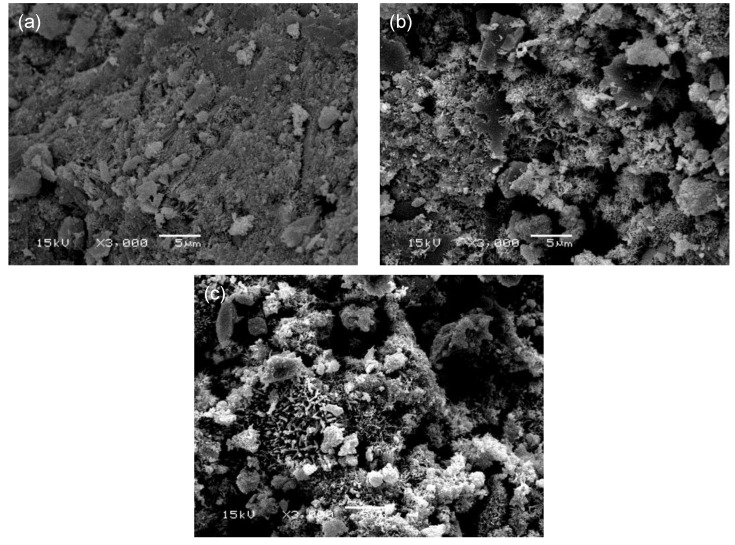
Scanning electron microscope micrographs of (**a**) OPC, (**b**) OPC-αHH20, and (**c**) PSC-αHH10 specimens at age of 28 days.

Meanwhile, as shown in Figure 10, ettringite was found in the αHH-replaced OPC-αHH20 at the age of 28 days, similar to the observation at the age of 3 days, but the effect on the suppression of the shrinkage strain was not as significant as that during the early age. However, the ettringite found in the αHH-replaced PSC-αHH10 specimen was significantly coarser than that in the OPC-αHH20 specimen, which has a significantly higher suppression effect on the shrinkage strain compared to the OPC-αHH20 specimen.

## 4. Conclusions

This study evaluated the effects of αHH, which is characterized by a quick initial setting time and expansion strain that occurs during its early age, on the setting, compressive strength, and shrinkage strain of cement mortar, and the following conclusions were made.

(1) The substitution of αHH into cement mortar decreases the initial setting time: the initial setting time clearly decreased with increasing replacement ratios of αHH. Meanwhile, when αHH was substituted in, the setting time of OPC was lower than that of PSC; however, the rate of decrease of the setting time of PSC was higher. This is due to the greater effect of αHH on the activation of the hydration reaction in PSC than in OPC.

(2) The compressive strength of cement mortar drastically decreased when αHH was substituted in and further decreased with increasing replacement ratios of αHH. It is clear that the substitution of αHH and the subsequent significant production of ettringite at an early age accelerated the hydration reaction; however, the residual ettringite that was not converted to monosulfate, even with progressing time, appeared to have negatively impacted the development of the compressive strength. Furthermore, the development of the compressive strength was more favorable when substituting αHH into PSC than into OPC.

(3) It was confirmed that expansion at the early age of αHH can decrease the shrinkage strain early in the aging of cement mortar. This is due to the growth pressure of the ettringite crystals formed from mixing with αHH. Meanwhile, the effect of substituting in αHH and suppressing the shrinkage strain in cement mortar was at a maximum at the early age of the material, and the production of ettringite was higher in PSC than in OPC; therefore, the suppression of the shrinkage strain was more effective.

## Figures and Tables

**Table 1:** Physical properties of materials used in the experiment.

Materials (Sign)	Physical Properties
ordinary Portland cement (OPC)	Density: 3.12 g/cm^3^, Blaine: 3500 cm^2^/g
Portland blast-furnace slag cement (PSC)	Density: 3.05 g/cm^3^, Blaine: 4000 cm^2^/g
α-calcium sulfate hemihydrate (αHH)	Density: 2.72 g/cm^3^, Blaine: 1400 cm^2^/g
sand (S)	Density: 2.50 g/cm^3^, Absorption ratio: 1.00%

**Table 2:** Chemical compositions of materials used in the experiment.

Materials	Chemical Composition (%)								
	SiO_2_	Al_2_O_3_	Fe_2_O_3_	CaO	MgO	Na_2_O	K_2_O	SO_3_	Loss on Ignition
OPC	20.70	6.20	3.10	62.20	2.80	0.10	0.84	2.10	1.96
PSC	27.11	9.84	1.88	52.66	3.40	0.31	0.66	2.43	1.71
αHH	2.57	0.88	0.41	39.99	0.32	-	-	55.79	0.04

**Table 3:** Experimental plan.

Specimen ID	Experimental Variables and Level		Evaluation Items
	Cement Type	Rep. Ratio of αHH	
OPC	OPC	-	▪ Setting time ▪ Compressive strength ▪ Drying shrinkage ▪ X-ray diffraction analysis ▪ Quantitative X-ray diffraction analysis ▪ Scanning electron microscope micrograph
OPC-αHH10		10	
OPC-αHH20		20	
OPC-αHH30		30	
PSC	PSC	-	
PSC-αHH10		10	

**Table 4:** Setting times of αHH-replaced cement mortar.

Specimen ID	Initial Setting Time (min)	Final Setting Time (min)	R^2^, Coefficient of Determination
OPC	287	412	0.9605
OPC-αHH10	272	426	0.9789
OPC-αHH20	137	455	0.9497
OPC-αHH30	6	350	0.7818
PSC	352	521	0.8853
PSC-αHH10	284	493	0.9727

**Table 5:** Quantitative X-ray diffraction analysis of OPC, OPC-αHH20, and PSC-αHH10 specimens.

Phases (%)	OPC		OPC-αHH20		PSC-αHH10	
	3 Days	28 Days	3 Days	28 Days	3 Days	28 Days
C_3_S monoclinic	2.36	3.05	2.44	2.30	1.41	1.82
C_2_S beta	4.60	5.76	0.88	3.09	2.36	5.82
C_3_A cubic	-	0.02	-	0.13	-	-
C_3_A Na orthorhombic	0.55	-	1.85	1.46	1.11	1.13
C_4_AF	1.75	2.95	2.70	3.16	1.84	1.90
Periclase	0.38	0.42	0.27	0.88	0.26	0.30
Lime	0.12	-	-	0.02	0.02	-
Arcanite	0.35	0.30	0.39	1.03	0.12	0.36
Gypsum	0.44	0.61	0.07	1.47	0.76	0.48
Bassanite	-	0.09	0.09	-	0.12	0.12
Calcite	0.27	2.58	0.85	3.73	0.55	3.07
Quartz	83.85	80.38	86.08	79.12	88.22	80.26
Portlandite	3.03	2.10	1.07	0.93	1.14	0.59
Ettringite	2.30	1.74	3.30	2.67	5.10	4.15
